# Successful Breast Conservation After Neoadjuvant Chemotherapy in Lobular Breast Cancer: The Role of Menopausal Status in Response to Treatment

**DOI:** 10.1245/s10434-023-14075-1

**Published:** 2023-08-10

**Authors:** Kirithiga Ramalingam, Elle Clelland, Harriet Rothschild, Firdows Mujir, Helena Record, Mandeep Kaur, Rita A. Mukhtar

**Affiliations:** 1 University of California, San Francisco, CA USA; 2 Department of Surgery, Carol Franc Buck Breast Care Center, San Francisco, CA USA

## Abstract

**Background:**

While neoadjuvant chemotherapy (NAC) has been shown to increase rates of breast conservation surgery (BCS) for breast cancer, response rates in invasive lobular carcinoma (ILC) appear lower than other histologic subtypes. Some data suggest higher response rates to NAC in premenopausal versus postmenopausal patients, but this has not been studied in ILC. We evaluated the rates of successful BCS after NAC in patients with ILC stratified by menopausal status.

**Patients and Methods:**

We analyzed data from a single-institution cohort of 666 patients with stage I–III hormone receptor positive HER-2 negative ILC. We used *t*-tests, chi-squared tests, and multivariable logistic regression to investigate rates of NAC use, attempted BCS, and associations between NAC and successful BCS by menopausal status.

**Results:**

In 217 premenopausal and 449 postmenopausal patients, NAC was used more often in the premenopausal group (15.2% vs. 9.8%, respectively, *p* = 0.041). Among those who attempted breast conservation (51.3% of pre- and 64.8% of postmenopausal cohorts), NAC was not associated with successful BCS in either group. Interestingly, for postmenopausal patients, receipt of NAC was significantly associated with increased rates of completion mastectomy in those who had positive margins at the first attempt at BCS.

**Conclusion:**

NAC was not associated with successful BCS in either premenopausal or postmenopausal patients with ILC. Although premenopausal patients were more likely to receive NAC, these data suggest that menopausal status may not be a good predictor of response to chemotherapy. Better predictors of response and more efficacious treatment for patients with ILC are needed.

The role of neoadjuvant chemotherapy (NAC) in increasing rates of breast conserving surgery (BCS) for patients with breast cancer has been well established in prospective randomized trials.^1–6^ However, response rates to NAC are influenced by tumor biology and other factors like tumor histology.^7^ For example, triple negative [estrogen receptor (ER) negative, progesterone receptor (PR) negative, and human epidermal growth factor-2 (HER-2) negative] and HER-2 positive tumors have higher rates of pathologic complete response (pCR) after NAC than hormone receptor (HR) positive HER-2 negative tumors.^8,9^ Additionally, many studies including a large meta-analysis show that tumors with lobular histology have lower response rates to NAC, resulting in lower rates of successful BCS.^10^ Invasive lobular carcinoma (ILC) is the second most common histologic type of breast cancer after invasive ductal carcinoma (IDC), accounting for 10–15% of breast cancer cases.^11^ Possibly due to its more diffuse growth pattern and the lower sensitivity of standard imaging tools, ILC presents at higher stages than IDC, and is known to have higher rates of positive margins at surgical excision, with the need for more completion mastectomies (unsuccessful BCS), as well as axillary dissections.^12,13^

While response rates to NAC are lower in those with ILC, there is still a subset of patients who do have pCR or partial response, highlighting the importance of personalizing treatment.^10,14,15^ Interestingly, some studies show that response to NAC may vary by menopausal status among patients with luminal breast cancer, with higher response rates in premenopausal patients compared with postmenopausal patients.^16,17^ This question has not been addressed specifically in those with ILC, where overall response rates are low. We therefore investigated whether NAC would be associated with higher rates of successful BCS in premenopausal patients with ILC compared with postmenopausal patients with ILC. Additionally, we sought to determine whether menopausal status influences which factors are associated with the timing of chemotherapy (neoadjuvant vs. adjuvant) in a single institution cohort of patients with ILC.

## Patients and Methods

**Fig. 1:**
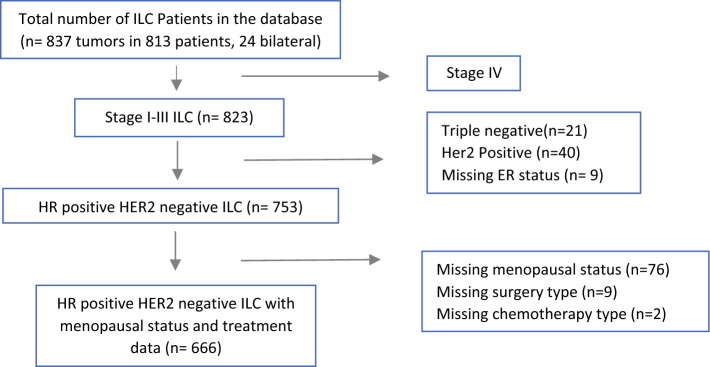
Patient selection flow chart. *ILC* invasive lobular carcinoma, *ER* estrogen receptor, *HR* hormone receptor, *HER-2* human epidermal growth factor receptor-2

We retrospectively analyzed data from a prospectively maintained institutional database containing clinicopathologic and treatment data for all consecutive patients who underwent surgery for ILC of the breast at our institution between 1992 and 2022 (Fig. 1). The study population was restricted to those with HR positive (ER or PR positive) and HER-2 negative disease. ER and PR status were considered positive if at least 1% of cells stained positively in immunohistochemistry (IHC); HER-2 status was considered positive with 3+ in IHC or positive in routine fluorescence in situ hybridization. Those with stage IV disease and missing data on HR status, receipt of chemotherapy, or menopausal status were excluded.

Clinicopathologic data including age, body mass index (BMI), pathologic tumor size, number of positive lymph nodes, stage of the disease, presence of multifocality, receptor subtype, grade, Ki67 index, presence of lymphovascular invasion (LVI), and presence of pleomorphic subtype were abstracted from source documents, including pathology reports, imaging reports, and operative reports. Details of systemic therapy (NAC vs. adjuvant chemotherapy) and menopausal status were abstracted from medical oncology notes. We report pathologic stage according to the 7th edition of the American Joint Commission on Cancer, with stage after NAC being “yp.” Surgical margins of “no ink on tumor” or wider were considered negative. Patients who received mastectomy after an attempt at BCS were considered to have undergone completion mastectomy; those who did not undergo completion mastectomy were classified as having successful BCS. The primary predictor variable for successful BCS was the receipt of NAC.

Stata 17 (Stata Corp. LLC, College Station, TX, USA) was used for statistical analyses. We used chi-squared tests for categorical variables and *t*-tests with one way analysis of variance for continuous variables to assess differences between pre- and postmenopausal women, and for univariable analysis of factors associated with NAC among pre- and postmenopausal women. We used a multivariable logistic regression model by forward selection method to evaluate the odds of completion mastectomy stratified by menopausal status among those who received chemotherapy; predictors included receipt of NAC, tumor size, number of positive lymph nodes, and margin status. Results are reported as odds ratios (OR), with 95% confidence intervals (CI), and two-sided *p* < 0.05 being considered significant. This study was approved by the institutional review board.

## Results

The study cohort comprised 666 cases of HR positive HER-2 negative ILC, of whom 449 (67.4%) were postmenopausal and 217 (32.6%) were premenopausal (Table 1). Overall, 226 (33.9%) patients received chemotherapy; of those 34.1% received NAC. Premenopausal patients differed from postmenopausal patients regarding age, BMI, stage, tumor biology, and treatment received. Average age was 47.2 years and 64.7 years in pre- and postmenopausal patients, respectively (*p* < 0.001), and premenopausal patients were significantly more likely to have a BMI in the normal range (62.4% vs. 46.7%, respectively, *p* < 0.01). When compared to postmenopausal patients, premenopausal patients were less likely to have early-stage disease (31.8% stage I vs. 41.9% stage I, *p* = 0.039), and PR negative disease (2.9% vs. 18.2%, *p* < 0.001). Chemotherapy use was significantly more common among premenopausal patients (46.1% vs. 28.1%, *p* < 0.001). Use of NAC was significantly more common among pre-menopausal patients compared to postmenopausal patients (15.2% vs. 9.8%, *p* = 0.041). Additionally, premenopausal patients were significantly less likely to attempt BCS as their first operation compared with postmenopausal patients (51.2% vs. 64.8%, respectively, *p* = 0.001).

**Table 1:** Clinicopathological and treatment characteristics of the study cohort by menopausal status

	Total *N* = 666 (%)	Premenopausal *N* = 217 (%)	Postmenopausal *N* = 449 (%)	*p* value
Mean age $\pm$ SD	59 ± 11.9	47.2 ± 4.9	64.7 ± 9.9	< 0.001
BMI				
18.5–25	325 (51.8)	126 (62.4)	199 (46.7)	<0.001
25–30	178 (28.3)	52 (25.7)	126 (29.6)	
≥ 30	125 (19.9)	24 (11.9)	101 (23.7)	
Stage				
I	257 (38.6)	69 (31.8)	188 (41.9)	0.039
II	293 (44.0)	108 (49.8)	185 (41.2)	
III	116 (17.4)	40 (18.4)	76 (16.9)	
Tumor size (mean ± SD, cm)	3.2 $\pm$ 2.9	3.6 $\pm$ 3.3	3.1 $\pm$ 2.7	0.016
Number of positive lymph nodes				
0	446 (67.8)	133 (61.6)	313 (70.8)	0.058
1–3	142 (21.6)	56 (25.9)	86 (19.5)	
> 3	70 (10.6)	27 (12.5)	43 (9.7)	
Tumor multifocality	211 (32.5)	85 (40.3)	126 (28.7)	0.003
Receptor subtype				
ER+ PR+ HER-2−	558 (86.8)	204 (97.1)	354 (81.7)	< 0.001
ER+ PR− HER2−	85 (13.2)	6 (2.9)	79 (18.2)	
Grade				
1	176 (27.1)	58 (27.8)	118 (26.8)	0.943
2	443 (68.3)	142 (67.9)	301 (68.4)	
3	30 (4.6)	9 (4.3)	21 (4.8)	
Ki67 (mean ± SD, percentage)	13.13 ± 9.4	12.09 ± 9.4	15.32 ± 14.9	0.016
LVI	41 (6.3)	13 (6.2)	28 (6.4)	0.944
Pleomorphic	56 (8.4)	20 (9.2)	36 (8)	0.601
Any chemotherapy	226 (33.9)	100 (46.1)	126 (28.1)	< 0.001
Neoadjuvant chemotherapy	77 (11.6)	33 (15.2)	44 (9.8)	0.041
Local therapy				
BCS	90 (13.7)	8 (3.7)	82 (18.6)	< 0.001
BCS + RT	234 (35.6)	73 (33.6)	161 (36.5)	
Mastectomy	231 (35.1)	84 (38.7)	147 (33.3)	
Mastectomy + RT	103 (15.7)	52 (24.0)	51 (11.6)	

*SD* Standard deviation, *BMI* Body mass index, *ER* Estrogen receptor, *PR* Progesterone receptor, *HER-2* Human epidermal growth factor receptor-2, *LVI* Lymphovascular invasion, *BCS* Breast conservation surgery, *RT* Radiation therapy

### Rates of Completion Mastectomies by Receipt of NAC and Menopausal Status

The majority of patients in this study (60.4%, *n* = 402) underwent BCS as their first operation. Of those, 74 (18.4%) subsequently had completion mastectomy. In total, 328 (49.3%) ultimately had successful BCS, with the remaining 338 (50.8%) having mastectomy.

**Fig. 2:**
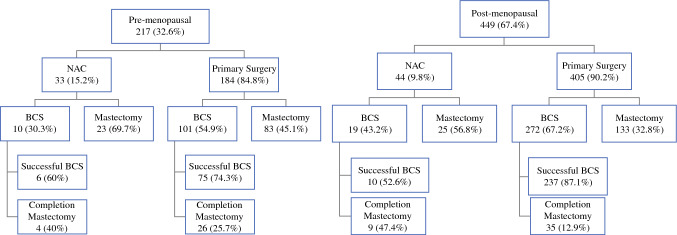
Rates of successful bcs by menopausal status. *NAC* neoadjuvant chemotherapy, *BCS* breast conservation surgery

Among the 217 premenopausal patients, 111 (51.2%) underwent BCS as their first operation, including 58.9% of those with T1 tumors, 63.8% of those with T2 tumors, and 21.8% of those with T3 tumors (Fig. 2). Overall, those receiving NAC were less likely to undergo attempted BCS compared with those who did not receive NAC (30.3% vs. 54.9%, *p* = 0.009). This remained true for those with T1 and T2 tumors; however, among those with T3 tumors, those receiving NAC were more likely to attempt BCS, although this did not reach statistical significance (30.8% vs. 19.1%, *p* = 0.371). In the premenopausal patients who had attempted BCS, 9% received NAC. Success of BCS was not associated with receiving NAC on univariable analysis; completion mastectomy rates were 25.7% in those without NAC, and 40.0% in those who received NAC (*p* > 0.05). In a multivariable logistic regression model including premenopausal patients who received chemotherapy and had attempted BCS, NAC was not significantly associated with lower odds of completion mastectomy (OR 0.88, 95% CI 0.11–7.03). However, factors associated with significantly increased odds of completion mastectomy were larger tumor size (OR 1.66 for every 1 cm increase in size, 95% CI 1.01–2.72) and positive margins at attempted BCS (OR 21.46, 95% CI 2.21–208.71) (Table 3).

Among the 449 postmenopausal patients, 291 (64.8%) underwent BCS as their first operation, including 78.5% of those with T1 tumors, 60.1% of those with T2 tumors, and 39.8% of those with T3 tumors (Fig. 2). Those who received NAC were significantly less likely to attempt BCS as the first operation compared with those who did not receive NAC (43.2% vs. 67.2%, *p* = 0.002). This was true for those with T1 tumors, but there was no difference in rates of attempted BCS by receipt of NAC in those with T2 or T3 tumors. Of the 291 postmenopausal patients who first underwent BCS, 6.5% had NAC. For those who did not receive NAC, the completion mastectomy rate was 12.9%, compared with 47.4% in those receiving NAC (*p* < 0.001). Interestingly, in those with positive margins after the first attempt at BCS, patients who did not receive NAC were significantly less likely to have completion mastectomy as their second operation compared with those who did receive NAC (28.8% vs. 61.5%, *p* = 0.023). In patients who did not receive NAC and had positive margins after attempted BCS, the majority (71.2%) had reexcision as a second operation as opposed to completion mastectomy. In a multivariable logistic regression model including postmenopausal patients who received chemotherapy and had attempted BCS, NAC was not significantly associated with lower odds of completion mastectomy (OR 2.62, 95% CI 0.71–9.68, Table 2).

**Table 2:** Multivariable logistic regression models predicting completion mastectomy in pre- and postmenopausal women who attempted BCS and received chemotherapy

	Premenopausal *N* = 45			Postmenopausal *N* = 65		
	OR	95% CI	*p* value	OR	95% CI	*p* value
Chemotherapy						
Neoadjuvant versus adjuvant	0.88	0.11–7.03	0.903	2.62	0.71–9.68	0.148
Mean tumor size (cm)	1.66	1.01–2.72	0.045	1.02	0.74–1.40	0.912
Mean positive nodes	0.96	0.78–1.17	0.671	0.99	0.90–1.11	0.968
Margin status						
Positive versus negative	21.46	2.21–208.71	0.008	2.36	0.62–9.04	0.209

### Factors Associated with Receipt of NAC in Pre- and Postmenopausal Patients

While NAC was not common in these patients with ILC, there were factors associated with its use in both pre- and postmenopausal women. In the premenopausal cohort, 100 (46.1%) patients received chemotherapy, with 33.0% of those receiving NAC and the remaining 67.0% receiving adjuvant chemotherapy. Those receiving NAC were significantly younger and had a higher mean tumor Ki67 compared with those receiving adjuvant chemotherapy (Table 3). Those with multifocal tumors were significantly less likely to receive NAC than those without multifocality (18.2% vs. 46.2%, respectively, *p* = 0.004). There was no association between receptor subtype, tumor grade, presence of lymphovascular invasion, pleomorphic status, BMI, or stage between those who received adjuvant versus neoadjuvant chemotherapy.

**Table 3:** Factors associated with timing of chemotherapy by menopausal status

	Premenopausal			Postmenopausal		
	Adjuvant chemotherapy *N* = 67 (%)	NAC *N* = 33 (%)	*p* value	Adjuvant chemotherapy *N* = 82 (%)	NAC *N* = 44 (%)	*p* value
Mean age ± SD	47.2 ± 4.7	44.9 ± 5.6	0.029	60.9 ± 8.6	57.2 ± 8.3	0.020
BMI						
18.5–25	36 (69.2)	16 (30.8)	0.453	34 (61.8)	21 (38.2)	0.063
25–30	16 (57.1)	12 (42.9)		18 (54.6)	15 (45.5)	
≥ 30	11 (73.3)	4 (26.7)		26 (81.3)	6 (18.8)	
Stage						
I	10 (58.8)	7 (41.2)	0.536	16 (72.7)	6 (27.3)	0.312
II	36 (72.0)	14 (28.0)		35 (58.3)	25 (41.7)	
III	21 (63.6)	12 (36.4)		31 (70.5)	13 (29.6)	
Tumor size (mean ± SD, cm)	4 ± 3.1	4.8 ± 4.2	0.035	3.81 ± 2.6	5.18 ± 3.6	0.016
Number of positive lymph nodes (mean ± SD)	2.9 ± 5.3	3.7 ± 5.4	0.463	3.68 ± 7.3	2 ± 3.3	0.150
Tumor multifocality	36 (81.8)	8 (18.8)	0.004	31 (68.9)	14 (31.1)	0.529
Receptor Subtype						
ER+ PR+ HER-2−	61 (67.0)	30 (32.9)	0.481	65 (65.66)	34 (34.34)	0.876
ER+ PR− HER-2−	2(50.0)	2 (50.0)		16 (64.00)	9 (36.00)	
Grade						
1	12 (48.0)	13 (52.0)	0.059	18 (60.0)	12 (40.0)	0.351
2	47 (73.4)	17 (26.6)		53 (65.4)	28 (34.6)	
3	4 (80.0)	1 (20.0)		10 (83.3)	2 (16.7)	
Ki67 (mean ± SD, percentage)	12 ± 9.9	23.4 ± 4.9	0.033	13.7 ± 0.8	18 ± 10.9	0.154
LVI	5 (55.6)	4 (44.4)	0.427	13 (68.4)	6 (31.6)	0.740
Pleomorphic	10 (76.9)	3 (23.1)	0.415	11 (73.3)	4 (26.7)	0.475

*SD* Standard deviation, *BMI* Body mass index, *ER* Estrogen receptor, *PR* Progesterone receptor, *HER-2* Human epidermal growth factor receptor-2, *LVI* Lymphovascular invasion

In the postmenopausal cohort, 126 (28.1%) of patients received chemotherapy, with 34.9% of those receiving NAC and the remaining 65.1% receiving it adjuvantly. Those receiving NAC were significantly younger and had larger tumor size compared to those receiving adjuvant chemotherapy (Table 3). There was no association with tumor multifocality, Ki67, receptor subtype, tumor grade, presence of lymphovascular invasion, pleomorphic status, BMI, or stage and timing of chemotherapy.

## Discussion

Because patients with ILC are diagnosed at higher stage, identifying predictors of responsiveness to therapy for patients with this tumor type is especially important to help improve surgical outcomes.^18^ Although the preponderance of data show that pathologic complete response rates are lower in ILC than IDC, whether NAC increases rates of successful BCS in those with ILC is less clear.^19,20^ Some studies show no differences in rates of BCS after NAC for those with ILC, while others show that NAC improves the likelihood of being a suitable candidate for BCS and achieving successful BCS for those who opt for it.^21–23^ Comparing studies can be challenging as some studies include ER negative and HER-2 positive ILC, which have been shown to have higher response rates to NAC than the more common ER positive HER-2 negative cases.^24^ Additionally, determining candidacy for BCS in patients with ILC may be challenging given decreased sensitivity of preoperative imaging.^25,26^ Similarly, whether a patient or provider will attempt BCS depends on multiple factors, including genetic testing results and patient preference. Regardless, the available data suggest that NAC may increase the chance of successful BCS for a subset of patients with ILC.

We evaluated whether examining surgical outcomes by menopausal status would give insight into the relationship between NAC and successful BCS in those with ILC. Some literature suggest higher response rates to NAC in premenopausal women with breast cancer compared with postmenopausal women, but the impact of menopausal status on response rates to NAC in ILC specifically is unknown.^16,17^ In our study, we did not find an association between NAC and successful BCS in either the pre- or postmenopausal cases. Patients who had NAC were in general less likely to attempt BCS; the only exception to this was among premenopausal patients with T3 ILC, where attempting BCS was more common in patients receiving NAC, suggesting an attempt to downstage tumors to optimize surgical outcomes. However, we did not identify any subsets where NAC was associated with higher rates of successful BCS.

While NAC has been associated with increased rates of successful BCS for breast cancer overall, it is also utilized more often in higher risk breast cancer, where patient preference may lead to higher rates of mastectomies at the first operation. For example, in both the pre- and postmenopausal cohorts’ receipt of NAC was associated with a significantly younger age at diagnosis. Multiple studies show higher rates of mastectomy than BCS in younger women. Our results may reflect this, making it harder to identify associations between NAC and improved candidacy for BCS. Additionally, the mean tumor size in the patients receiving NAC was 4.8 cm and 5.2 cm in the pre- and postmenopausal cohorts, respectively. As such, these patients may not have been considered good candidates for BCS, since many studies of BCS exclude patients with tumors > 4 cm in size.^27^ We chose to evaluate pathologic tumor size, as this would most closely approximate posttreatment tumor size, which is typically the basis for surgical decision making. However, discordance between clinical assessment of posttreatment tumor size and pathologic tumor size in patients with ILC increases the challenge of surgical planning.^28^

Because patients undergoing NAC had large tumors, we adjusted for tumor size when evaluating the association between NAC and successful BCS. In this multivariable model, we found no association between NAC and successful BCS. Of note, we restricted this model to patients who received chemotherapy to minimize differences between those who received chemotherapy and those who did not due to differences in tumor biology. We found that larger tumor size and positive margins were associated with significantly higher odds of completion mastectomy in the premenopausal cohort. This differed from the postmenopausal cohort, where neither tumor size nor margin status were associated with completion mastectomy. It is possible that tumor size was only associated with NAC in premenopausal women because of a potential difference in rates of desired BCS. Interestingly, in the postmenopausal cohort, we found that when positive margins were encountered in the subset of patients who received NAC, the next operation was much more likely to be completion mastectomy instead of an attempt at reexcision compared with those who had positive margins without NAC. We do not know the reasons for this differential management, but we hypothesize that positive margins after NAC were perhaps viewed as indicative of treatment resistance that might warrant more aggressive management.

Regarding patient selection for NAC, our study is limited by its retrospective nature and inherent selection bias, as well as the absence of data on preoperative imaging modalities and genetic testing data. We found that among premenopausal women, younger age and higher Ki67 were both associated with receiving NAC. For postmenopausal women, younger age was also associated with NAC, as was larger tumor size. This practice pattern is consistent with data suggesting increased response rates to NAC in tumors with higher Ki67.^29^ Additionally, we previously found that younger age and larger tumor size were significantly associated with receipt of preoperative systemic therapy in patients with ILC in the National Cancer Database.^30^ However, the absence of a relationship between grade, pleomorphic status, and nodal status with receiving NAC in this HR positive HER-2 negative population may reflect the lack of clear indicators of benefit from NAC in this patient population.

## Conclusion

Overall, we found that menopausal status does not appear to be a good predictor of response to NAC in patients with HR positive HER-2 negative ILC, as determined by rates of successful BCS. However, premenopausal patients were much more likely to receive NAC than postmenopausal patients with ILC. These findings highlight the need to identify better indicators of response to chemotherapy in ILC patients, particularly in the preoperative setting, and the need for potential alternative systemic therapies that may have greater efficacy in this tumor type.

## References

[1] van der Hage JA, van de Velde CJ, Julien JP, Tubiana-Hulin M, Vandervelden C, Duchateau L (2001). Preoperative chemotherapy in primary operable breast cancer: results from the European Organization for Research and Treatment of Cancer trial 10902. J Clin Oncol..

[2] Mauri D, Pavlidis N, Ioannidis JPA (2005). Neoadjuvant versus adjuvant systemic treatment in breast cancer: a meta-analysis. J Natl Cancer Inst..

[3] Boughey JC, Peintinger F, Meric-Bernstam F (2006). Impact of preoperative versus postoperative chemotherapy on the extent and number of surgical procedures in patients treated in randomized clinical trials for breast cancer. Ann Surg..

[4] Rastogi P, Anderson SJ, Bear HD (2008). Preoperative chemotherapy: updates of national surgical adjuvant breast and bowel project protocols B-18 and B-27. J Clin Oncol..

[5] Barry PA, Schiavon G (2015). Primary systemic treatment in the management of operable breast cancer: best surgical approach for diagnosis, biological evaluation, and research. J Natl Cancer Inst Monogr..

[6] Early Breast Cancer Trialists’ Collaborative Group (EBCTCG) (2018). Long-term outcomes for neoadjuvant versus adjuvant chemotherapy in early breast cancer: meta-analysis of individual patient data from ten randomised trials. Lancet Oncol..

[7] Chica-Parrado MR, Godoy-Ortiz A, Jiménez B, Ribelles N, Barragan I, Alba E (2020). Resistance to neoadjuvant treatment in breast cancer: clinicopathological and molecular predictors. Cancers..

[8] Untch M, Möbus V, Kuhn W (2009). Intensive dose-dense compared with conventionally scheduled preoperative chemotherapy for high-risk primary breast cancer. J Clin Oncol..

[9] Esserman LJ, Berry DA, DeMichele A (2012). Pathologic complete response predicts recurrence-free survival more effectively by cancer subset: results from the I-SPY 1 TRIAL—CALGB 150007/150012, ACRIN 6657. J Clin Oncol..

[10] O’Connor DJ, Davey MG, Barkley LR, Kerin MJ (2022). Differences in sensitivity to neoadjuvant chemotherapy among invasive lobular and ductal carcinoma of the breast and implications on surgery-A systematic review and meta-analysis. Breast..

[11] Li CI, Anderson BO, Daling JR, Moe RE (2003). Trends in incidence rates of invasive lobular and ductal breast carcinoma. JAMA..

[12] Lopez JK, Bassett LW (2009). Invasive lobular carcinoma of the breast: spectrum of mammographic, US, and MR imaging findings. RadioGraphics..

[13] Piper ML, Wong J, Fahrner-Scott K (2019). Success rates of re-excision after positive margins for invasive lobular carcinoma of the breast. NPJ Breast Cancer..

[14] Riba LA, Russell T, Alapati A, Davis RB, James TA (2019). Characterizing response to neoadjuvant chemotherapy in invasive lobular breast carcinoma. J Surg Res..

[15] Tsung K, Grobmyer SR, Tu C, Abraham J, Budd GT, Valente SA (2018). Neoadjuvant systemic therapy in invasive lobular breast cancer: is it indicated?. Am J Surg..

[16] da Silva LR, Vargas RF, Shinzato JY, Derchain SFM, Ramalho S, Zeferino LC (2019). Association of menopausal status, expression of progesterone receptor and Ki67 to the clinical response to neoadjuvant chemotherapy in luminal breast cancer. Rev Bras Ginecol Obstet..

[17] Yamamoto Y, Yamaguchi R, Fujiki Y, Ibusuki M, Murakami K, Iwase H (2012). Association of response to neoadjuvant chemotherapy (NAC) in premenopausal patients with hormone receptor-positive early breast cancer with chemotherapy-induced ovarian function suppression by NAC. JCO..

[18] Pestalozzi BC, Zahrieh D, Mallon E (2008). Distinct clinical and prognostic features of infiltrating lobular carcinoma of the breast: combined results of 15 International Breast Cancer Study Group clinical trials. J Clin Oncol..

[19] Lips EH, Mulder L, de Ronde JJ (2012). Neoadjuvant chemotherapy in ER+ HER2- breast cancer: response prediction based on immunohistochemical and molecular characteristics. Breast Cancer Res Treat..

[20] von Minckwitz G, Untch M, Blohmer JU (2012). Definition and impact of pathologic complete response on prognosis after neoadjuvant chemotherapy in various intrinsic breast cancer subtypes. JCO..

[21] Boughey JC, Wagner J, Garrett BJ (2009). Neoadjuvant chemotherapy in invasive lobular carcinoma may not improve rates of breast conservation. Ann Surg Oncol..

[22] Fitzal F, Mittlboeck M, Steger G (2012). Neoadjuvant chemotherapy increases the rate of breast conservation in lobular-type breast cancer patients. Ann Surg Oncol..

[23] Truin W, Vugts G, Roumen RMH (2016). Differences in response and surgical management with neoadjuvant chemotherapy in invasive lobular versus ductal breast cancer. Ann Surg Oncol..

[24] Lips EH, Mukhtar RA, Yau C (2012). Lobular histology and response to neoadjuvant chemotherapy in invasive breast cancer. Breast Cancer Res Treat..

[25] López-Narváez RA, Garza-Montemayor ML, Garza-García NL (2012). Detection of invasive breast lobular carcinoma by image analysis: comparison between mammography and ultrasound. Ginecol Obstet Mex..

[26] Weaver O, Yang W (2020). Imaging of breast cancers with predilection for nonmass pattern of growth: invasive lobular carcinoma and DCIS—Does imaging capture it all?. Am J Roentgenol..

[27] Abel MK, Brabham CE, Guo R (2021). Breast conservation therapy versus mastectomy in the surgical management of invasive lobular carcinoma measuring 4 cm or greater. Am J Surg..

[28] Shaikh A, Tariq MU, Khan SM (2021). Concordance Between clinical and pathological response assessment after neo-adjuvant chemotherapy in patients with invasive lobular carcinoma. Cureus..

[29] Kim KI, Lee KH, Kim TR, Chun YS, Lee TH, Park HK (2014). Ki-67 as a predictor of response to neoadjuvant chemotherapy in breast cancer patients. J Breast Cancer..

[30] Mukhtar RA, Hoskin TL, Habermann EB, Day CN, Boughey JC (2021). Changes in management strategy and impact of neoadjuvant therapy on extent of surgery in invasive lobular carcinoma of the breast: analysis of the National Cancer Database (NCDB). Ann Surg Oncol..

